# XatA, an AT-1 autotransporter important for the virulence of *Xylella fastidiosa* Temecula1

**DOI:** 10.1002/mbo3.6

**Published:** 2012-03

**Authors:** Ayumi Matsumoto, Sherry L Huston, Nabil Killiny, Michele M Igo

**Affiliations:** 1 Department of Microbiology, University of CaliforniaDavis, California, 95616; 2 Citrus Research and Education Center, Department of Entomology and Nematology, University of FloridaIFAS, 700 Experiment Station Road, Lake Alfred, Florida 33850

**Keywords:** Adhesin, biofilm, sharpshooter, type V secretion, virulence factor

## Abstract

*Xylella fastidiosa* Temecula1 is the causative agent of Pierce's disease of grapevine, which is spread by xylem-feeding insects. An important feature of the infection cycle is the ability of *X. fastidiosa* to colonize and interact with two distinct environments, the xylem of susceptible plants and the insect foregut. Here, we describe our characterization of XatA, the *X. fastidiosa* autotransporter protein encoded by PD0528. XatA, which is classified as an AT-1 (classical) autotransporter, has a C-terminal β-barrel domain and a passenger domain composed of six tandem repeats of approximately 50 amino acids. Localization studies indicate that XatA is present in both the outer membrane and membrane vesicles and its passenger domain can be found in the supernatant. Moreover, XatA is important for *X. fastidiosa* autoaggregation and biofilm formation based on mutational analysis and the discovery that *Escherichia coli* expressing XatA acquire these traits. The x*atA* mutant also shows a significant decrease in Pierce's disease symptoms when inoculated into grapevines. Finally, *X. fastidiosa* homologs to XatA, which can be divided into three distinct groups based on synteny, form a single, well-supported clade, suggesting that they arose from a common ancestor.

## Introduction

*Xylella fastidiosa* is a Gram-negative, endophytic bacterium, which is responsible for numerous economically important plant diseases, including Pierce's disease (PD) of grapevine (*Vitis vinifera*) (for reviews, see Hopkins and Purcell 2002; Chatterjee et al. 2008). This pathogen is transmitted from infected plants to susceptible plant species by xylem-feeding insects, such as spittlebugs and sharpshooters. Once inside the xylem, *X. fastidiosa* moves from the inoculation site into new xylem vessels, eventually forming a biofilm, which blocks the flow of sap within the grapevine. The resulting symptoms include irregular scorching of the leaf, separation of the leaf blade from the petiole (matchsticks), irregular lignification (green islands), shriveling of grape berries, and the eventual death of the vine. Although similar, the symptoms associated with PD are qualitatively and quantitatively distinct from symptoms resulting from water stress and are thought to be a consequence of the plant's response to bacterial invasion and the production of virulence factors by *X. fastidiosa* following colonization of the xylem tissue (Stevenson et al. 2005; Thorne et al. 2006).

Comparison of the *X. fastidiosa* Temecula1 genome to other bacterial pathogens has resulted in the identification of a number of potential virulence factors (Van Sluys et al. 2003; Chatterjee et al. 2008). One important category includes virulence determinants delivered to the bacterial cell surface through type V secretion systems, such as autotransporters (for reviews, see Henderson et al. 2004; Dautin and Bernstein 2007). The conventional or classical autotransporters (AT-1) possess an N-terminal passenger domain, which encodes the effector function of the mature protein, and a C-terminal β-barrel domain, which anchors the protein to the outer membrane (OM). Biochemical and crystallographic studies indicate that most AT-1 autotransporters are monomeric and the overall tertiary structure of their C-terminal domains is highly conserved, containing 12 transmembrane β-strands and an α-helix inside the β-barrel. The diversity among the AT-1 proteins can be found in their passenger domains. Functions associated with this domain include proteolytic activity, adherence, biofilm formation, intracellular motility, cytotoxic activity, or maturation of another virulence determinant. AT-1 proteins also possess an N-terminal signal sequence, which is responsible for their transport across the inner membrane and a C-terminal signature sequence, which facilitates their interaction with the BAM (β-barrel assembly machine) complex and their ultimate transport to the cell surface (Knowles et al. 2009). Finally, when expressed in a heterologous system such as *Escherichia coli*, many AT-1 proteins are properly localized to the cell surface and their passenger domains exhibit effector function. Indeed, this ability of AT-1 proteins has been exploited to study the properties of numerous proteins in a strategy known as autotransporter-mediated surface display (autodisplay) (Jose and Meyer 2007; van Bloois et al. 2011).

Based on genomic analysis, there are six members of the AT-1 autotransporter family in *X. fastidiosa* Temecula1. Here, we describe our characterization of XatA, the autotransporter protein encoded by PD0528. Localization studies indicate that XatA is found in both the OM and OM vesicles (OMVs) and its passenger domain is present on the cell surface. The released form of the XatA passenger domain can also be found in the supernatant. Moreover, mutational analysis, in combination with complementation analysis, indicates that XatA is important for autoaggregation and biofilm formation under laboratory conditions. Further support for this conclusion comes from the observation that *E. coli* cells expressing XatA exhibit new phenotypic properties, including autoaggregation and biofilm formation. Finally, XatA is required for the development of PD symptoms in grapevines. Together, these data demonstrate that XatA is an important virulence factor in *X. fastidiosa* Temecula1.

## Results

### Identification of the autotransporter XatA

To gain insight into how the protein composition of the cell surface influences pathogenicity, we initiated a study to identify the major OM proteins (OMPs) and to assign them to specific genes on the *X. fastidiosa* genome (Igo 2004). One of the identified OMPs, which is encoded by the PD0528 locus, exhibited features characteristic of AT-1 autotransporters (Henderson et al. 2004; Dautin and Bernstein 2007). Based on this homology, we named this protein XatA for *X. fastidiosa* autotransporter protein A.

**Figure 1:**
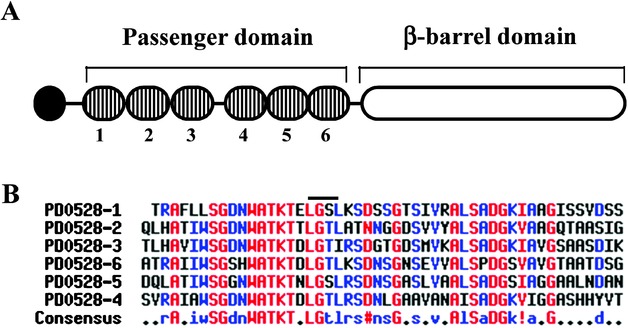
Structural features of XatA. (A). Diagram giving an overview of the structural features in XatA: the signal peptide (black), the six tandem repeats (vertical bars), and the β-barrel domain (white). The numbers identify the different tandem repeats, which are located at the following positions: 69–117 (PD0528-1), 120–169 (PD0528-2), 170–219 (PD0528-3), 236–285 (PD0528-4), 294–343 (PD0528-5), 344–393 (PD0528-6). (B). Alignment of the six repeats in the passenger domain. The six repeats were aligned by ClustalW2 (Chenna et al. 2003) and visualized by Multalin (Corpet 1988). Colors are used to identify the amount of conservation: high (red), low (blue), and neutral (black). In the consensus sequence, two additional symbols are used: # (D or N) and ! (I or V). The black bar above the alignment indicates the location of the LGxL motif.

The XatA protein is 733 amino acids in length and has a theoretical molecular mass of 76.5 kDa. Its putative domain structure is depicted schematically in Figure 1A. The C-terminal 256 residues show homology to the conserved β-barrel domain of AT-1 autotransporters. XatA is also predicted to contain two sequences that mediate its proper localization. Analysis of the sequence with SignalP (Emanuelsson et al. 2007) predicts that XatA has a potential N-terminal signal peptide with a signal peptidase cleavage site between residues A29 and N30. A cleaved signal peptide of ∼20–30 amino acids is present in most AT-1 autotransporters and is a common feature of proteins dependent on the Sec pathway for transport across the inner membrane (Dautin and Bernstein 2007). XatA also contains a potential C-terminal OMP signature sequence, which includes the conserved phenylalanine as the final amino acid (Struyve et al. 1991; Henderson et al. 2004). The presence of this sequence suggests that XatA is dependent on the BAM complex for its ultimate transport to the cell surface (Knowles et al. 2009).

The unique features of XatA are found in its passenger domain, which is composed of tandem repeats of a 50 amino acid motif. Computer analysis of XatA using secondary structural prediction programs and multiple alignment programs indicated that the XatA passenger domain contains six tandem repeats and that the similarity between the repeats is quite high. As shown in Figure 1B, each repeat contains blocks of strictly conserved amino acids and an LGxL repeat. Proteins having an LGxL repeat are predicted to form a β-propeller, a structure often involved in protein–protein interactions (Adindla et al. 2007).

### Subcellular localization of XatA

**Figure 2:**
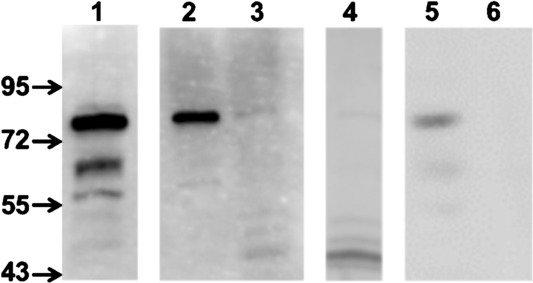
Subcellular localization of XatA and the XatA passenger domain. Proteins from the different fractions were separated on a 7.5% SDS-PAGE and subjected to Western analysis using antibodies against the XatA passenger domain (αRXatA): outer membrane (OM) (lane 1), OM vesicles (OMV) (lane 2), the secreted protein fraction (1 μl; lane 3), the secreted protein fraction (7 μl; lane 4). To determine the susceptibility of XatA to protease digestion, intact *Xylella fastidiosa* cells were treated with proteinase K and OM proteins (OMPs) were then isolated and subjected to Western analysis: OM from untreated cells (lane 5), OM from cells after proteinase K treatment (lane 6).

To determine the subcellular localization of XatA and the XatA passenger domain, a culture of *X. fastidiosa* Temecula1 was prepared and the cells were separated from the supernatant by centrifugation. The OM fraction was isolated from disrupted cells by sucrose density gradient centrifugation as previously described (Voegel et al. 2010); the OMVs and secreted protein fractions were obtained by filtration and serial centrifugation of the supernatant. The proteins in these fractions were then separated by sodium dodecyl sulfate-polyacrylamide gel electrophoresis (SDS-PAGE) and subjected to Western analysis using antibodies against the XatA passenger domain (αRXatA). As shown in Figure 2, a protein of ∼76 kDa was detected in the OM (lane1) and OMV fraction (lane 2). Based on the size, the XatA protein in these fractions has both its passenger domain and the autotransporter domain. In contrast, the secreted protein fraction contains a protein of ∼45 kDa (lanes 3, 4), which corresponds to the size of the XatA passenger domain. We next examined whether or not the XatA passenger domain is surface exposed using a proteinase K accessibility assay. Proteinase K, which is unable to diffuse across the OM of Gram-negative bacteria, will only cleave the surface proteins of intact bacteria. As shown in Figure 2, αRXatA recognized a protein that migrated at the size of the intact XatA protein (∼76 kDa) in the untreated sample (lane 5). However, this protein was missing in the proteinase K treated sample (lane 6). This is consistent with our hypothesis that the XatA passenger domain is exposed on the *X. fastidiosa* cell surface.

### Properties of the *xatA3* mutant in culture

**Figure 3:**
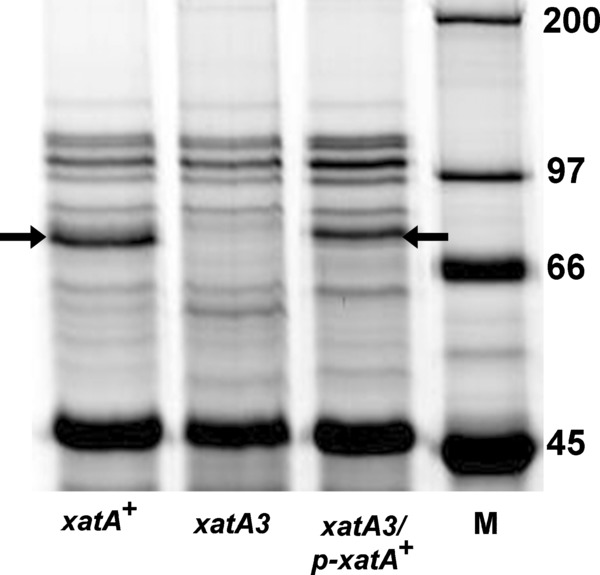
OM profile of the *xatA3* mutant. The OM proteins were separated on an 8% SDS-PAGE and then stained with SyproRuby: *xatA*^+^ (*X. fastidiosa* Temecula1), the *xatA3* mutant, (TAM103), and the *xatA* 3/p- *xatA*^+^ complementation strain (TAM103/pAM61). Lane M shows the molecular weight standard labeled in KDa. The identification of the bands indicated by the arrows as the XatA protein was confirmed by MALDI-TOF mass spectrometry (data not shown).

**Figure 4:**
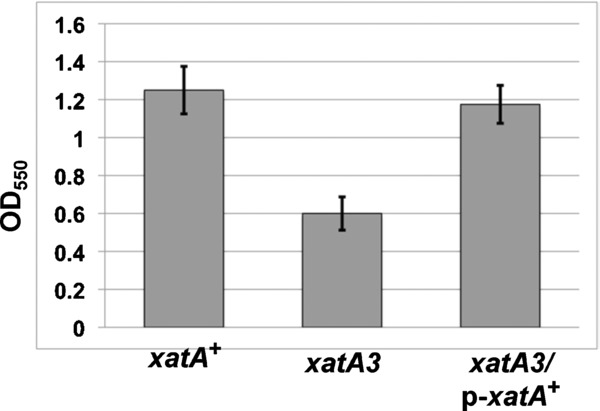
Impact of XatA on biofilm formation. Cells were grown in PD3 in 18-mm glass tubes for seven days without agitation: *xatA*^+^ (Temecula1), *xatA3* (TAM103), and *xatA* 3/p- *xatA*^+^ (TAM103/pAM61). Biofilms were stained with crystal violet and resuspended in 95% ethanol. The absorbance at 550 nm was then measured to quantify biofilm production.

To investigate the role of XatA in *X. fastidiosa* cell physiology and virulence, we generated a null mutation in the *xatA* gene by inserting a chloramphenicol-resistance gene cassette within the XatA open reading frame (ORF) (*xatA3*::Cm^r^) using site-directed gene disruption (Feil et al. 2003). This resulted in the *xatA3* mutant, TAM103 (Table 1). We also generated a strain for complementation analysis called *xatA3*/p-*xatA*^+^ (TAM103/pAM61). The plasmid pAM61 is a derivative of pBBR1MCS-5 (Kovach et al. 1995), which carries the wild-type *xatA* gene. We first examined the impact of the *xatA3*::Cm^r^ mutation on the OM protein profile. As shown in Figure 3, a protein band running at ∼76 kDa is present in the OM of *xatA*^+^ (wild type) and *xatA3*/p-*xatA*^+^, but is missing in the *xatA3* mutant. The identification of the ∼76 kDa band as XatA was confirmed by MALDI-TOF mass spectrometry (data not shown). The next step was to investigate the impact of the *xatA3*::Cm^r^ mutation on bacterial growth under laboratory conditions. The three strains had similar doubling times in liquid media and were not able to form a confluent lawn on plates, which is characteristic of wild-type *X. fastidiosa* strains (data not shown). We then examined their ability to form free-floating cell aggregates (autoaggregation) and a biofilm in static liquid media. In contrast to *xatA*^+^ and *xatA3*/p-*xatA*^+^ cells, the *xatA3* mutant formed smaller and less abundant aggregates (data not shown). Moreover, as shown in Figure 4, the amount of biofilm produced by the *xatA3* mutant was lower than wild type and it was possible to restore wild-type levels of biofilm formation to the *xatA3* mutant by introducing p-*xatA*^+^. Thus, the absence of XatA impacts the ability of *X. fastidiosa* to autoaggregate and to form a biofilm under laboratory conditions.

**Table 1:** Key bacterial strains and plasmids used in this study

Strains	Relevant genotype	Source
*Xylella fastidiosa* subsp. *fastidiosa*		
*xatA* ^+^ (Temecula1)	*xatA* ^+^	Guilhabert et al. (2001)
*xatA* ^+^ Cm^r^ (TAM22)	*xatA*^+^, NS1::Cm^r^	Matsumoto et al. (2009)
*xatA3* (TAM103)	*xatA3*::Cm^r^	This study
*xatA3* /p- *xatA*^+^ (TAM103/pAM61)	*xatA3*::Cm^r^ / *xatA*^+^, Gm^r^	This study
*Escherichia coli*		
UT5600	Δ(*ompT-fepC266)* Δ *ompP*	Elish et al. (1988); Kaufmann et al. (1994)
Plasmids		
Vector (pBBR1MCS-5)	Broad-host range cloning vector, Gm^r^	Kovach et al. (1995)
p- *xatA*^+^ (pAM61)	*xatA*^+^ (3.5 kb) in pBBR1MCS-5 *,* Gm^r^	This study

### Expression of XatA in *E. coli* results in autoaggregation and biofilm formation

**Figure 5:**
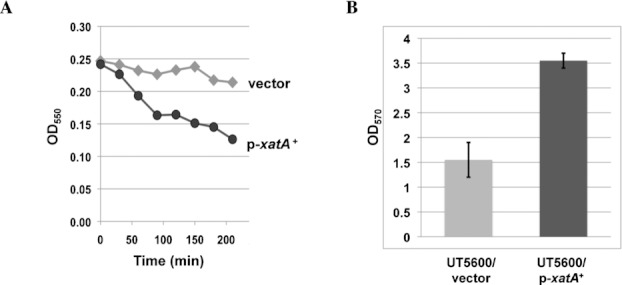
Heterologous expression of XatA in *Escherichia coli*. (A). Aggregation of XatA-expressing cells. Cells were vortexed for 10 sec and incubated statically for 3.5 h at room temperature. Samples were taken from the top of the tube at 30-min intervals and the absorbance at 550 nm was determined for UT5600 containing either the vector (pBBR1MCS-5) or p- *xatA*^+^ (pAM61). (B). Biofilm formation by XatA-expressing cells. Cells were grown in LB in 18-mm tubes for two days without agitation. Biofilms were stained with crystal violet and resuspended in 95% ethanol. The absorbance at 570 nm was then measured to quantify biofilm production.

Another method we used to examine the function of the XatA passenger domain was to express the protein in a heterologous system. For this analysis, p-*xatA*^+^ (pAM61) and the vector (pBBR1MCS-5) were introduced into UT5600, an *E. coli* strain deficient in the two OM proteases, OmpT and OmpP (Elish et al. 1988; Kaufmann et al. 1994). Western analysis and proteinase K accessibility studies using αRXatA confirmed that XatA is present on the UT5600/ p-*xatA*^+^ cell surface (data not shown). Moreover, unlike the control cells, cells expressing XatA exhibit autoaggregation (Fig. 5A) and are able to form a biofilm (Fig. 5B). The ability of XatA to confer new phenotypic properties to *E. coli* indicates that XatA is directly responsible for the observed traits.

### XatA impacts *X. fastidiosa* virulence

**Figure 6:**
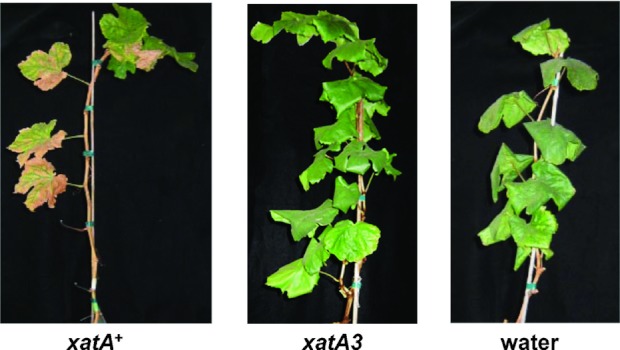
The *xatA3* mutant impacts *X. fastidiosa* virulence in grapevines. Greenhouse-grown grapevines (cv. Thompson seedless) were inoculated using the needle puncture method with one of the following: *xatA*^+^ (*X. fastidiosa* Temecula1), *xatA3* (TAM103), or water (mock infection). These photographs show representative vines 16 weeks after infection.

To determine the role of XatA in *X. fastidiosa* virulence, greenhouse-grown grapevine (cv. Thompson seedless) were inoculated by the needle puncture method (Hill and Purcell 1995) with one of the following: wild type, the *xatA3* mutant, or water (mock infection). After 16 weeks, plants inoculated with wild type developed symptoms characteristic of PD, including leaf scorch and match stick formation (Fig. 6). In contrast, the plants inoculated with either *xatA3* or water did not develop symptoms. We then prepared suspensions from petiole tissues taken from both symptomatic and asymptomatic plants at multiple points above the inoculation site and determined the size of the *X. fastidiosa* population as previously described (Matsumoto et al. 2009). For vines infected with the wild-type strain, *X. fastidiosa* could be recovered from petioles located at multiple points above inoculation site: 3.3 × 10^4^ colony-forming units (cfu) g^−1^ of tissue at 4 cm, 1.4 × 10^4^ cfu g^−1^ at 20 cm, and 1.2 × 10^4^ cfu g^−1^ at 40 cm. No bacterial colonies were recovered from mock-inoculated grapevines. Interestingly, fewer bacteria were recovered from the *xatA3*-infected vines. At 4 cm, only 8.8 × 10^3^ cfu g^−1^ were recovered and no bacteria were recovered at either 20 or 40 cm. These results suggest that the absence of XatA impacts both migration and the ability of *X. fastidiosa* to colonization the grapevine.

In an independent series of experiments, we compared the properties of vines infected with *xatA3*/p-*xatA*^+^ to *xatA3* or a wild-type strain carrying a chloramphenicol cassette at a neutral site, *xatA*^+^ Cm^r^ (TAM22) (Matsumoto et al. 2009). After 24 weeks, plants inoculated with the *xatA*^+^ Cm^r^ strain developed symptoms characteristic of PD, whereas plants inoculated with either *xatA3* or *xatA3*/p-*xatA*^+^ did not (data not shown). We then determined the size and properties of the *X. fastidiosa* population at multiple locations above the inoculation sites by plating onto PD3 plates. We also plated the bacteria recovered from the *xatA3*/p-*xatA*^+^-infected vines onto PD3 containing gentamicin to determine what percentage of the population had maintained the plasmid (pAM61). In contrast to the *xatA*^+^ Cm^r^ strain, approximately 10-fold fewer bacteria were recovered from *xatA3*-infected and *xatA3*/p-*xatA*^+^-infected vines at 12.5 cm from the inoculation site and no *X. fastidiosa* were recovered at 25 cm. Moreover, examination of bacteria recovered from *xatA3*/p-*xatA*^+^-infected vines indicated that the strain had lost the p-*xatA*^+^ plasmid. This is consistent with our previous work indicating that pBBR1MCS-5-derived plasmids are not retained in *X. fastidiosa* in the absence of selective pressure (Matsumoto et al. 2009).

**Figure 7:**
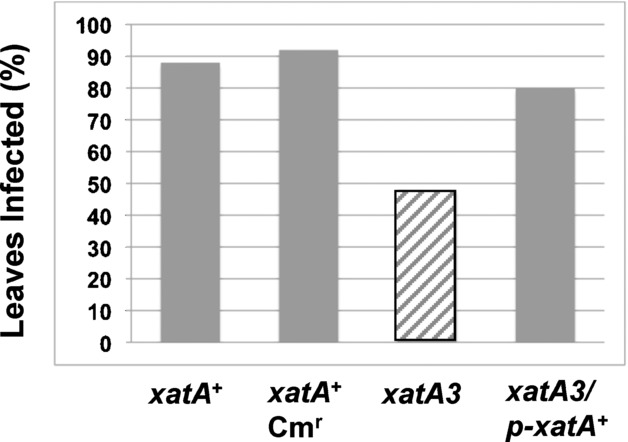
The impact of XatA on insect-mediated infection. Individual blue-green sharpshooters were allowed access to a sachet containing a diet solution and the indicated strain for 3 h: *xatA*^+^ (*X. fastidiosa* Temecula1), *xatA*^+^ Cm^r^ (TAM22), *xatA3* (TAM103), and *xatA* 3/p- *xatA*^+^ (TAM103/pAM61). The insects were then individually transferred to a single leaf on a grape host and allowed to feed for four days. After 12 weeks, the leaves were collected and the petioles were examined for the presence or absence of *X. fastidiosa*. The results with the *xatA3* mutant (diagonal bar) showed a significant difference (*P* < 0.05) when compared to those for other three strains (gray bars).

Finally, we examined the importance of XatA in the initial stages of infection by the insect vector. In this experiment, *xatA*^+^, *xatA*^+^ Cm^r^, *xatA3*, and *xatA3*/p-*xatA*^+^ were delivered to uninfected blue-green sharpshooters (*Graphocephala atropunctata*) using an artificial diet system (Killiny and Almeida 2009a; Killiny et al. 2012). The insects are then individually caged on a single leaf of a grape host and were allowed to feed for four days. After 12 weeks, the leaves were collected and examined for the presence of *X. fastidiosa*. Twenty-five replicates were performed for each strain. As shown in Figure 7, *X. fastidiosa* was present in 48% of the leaves exposed to sharpshooters fed with the diet solution containing *xatA3* compared to the 88% observed for *xatA*^+^. This difference was due to the absence of XatA and not due to the presence of the chloramphenicol-resistance gene based on the results with *xatA*^+^ Cm^r^ (92%). Moreover, *X. fastidiosa* was present in 80% of the leaves collected from the analysis with *xatA3*/p-*xatA*^+^, indicating that complementation of the *xatA3*::Cm^r^ mutation had occurred. Given the instability of *p-xatA*^+^ in the absence of selective pressure, the complementation data suggest that XatA plays a role early in the infection process. The next step will be to refine the role of XatA even further by examining its importance in attachment and colonization of the insect foregut directly.

### Proteins homologous to the XatA passenger domain

Homologs to the XatA passenger domain are present in all sequenced *X. fastidiosa* strains and a few other Gram-negative bacteria. The phylogenetic relationships between XatA and these proteins are shown in Figure S1. The tree revealed that the XatA homologs present in *X. fastidiosa* fall into one clade (the Xat family), which contains three distinct well-supported paralogous groups. Group I includes orthologs to XatA, which are present in all North American isolates of *X. fastidiosa*. Within the sequenced *X. fastidiosa* subsp. *fastidiosa* isolates that cause PD (strains Temecula1, M13, and EB92.1), the amino acid sequence of XatA is completely conserved. Orthologs to XatA are also present in the strain that causes oleander leaf scorch (*X. fastidiosa* subsp. *sandyi* Ann-1) and the strains that cause almond leaf scorch (*X. fastidiosa* subsp. *multiplex* strains Dixon and M12). Their assignment to Group I also has significant bootstrap support by synteny and by the high level of amino acid identity (96%). The main location of amino acid differences is in a region of low complexity between the passenger domain and the autotransporter domain.

In the South American isolate that causes citrus variegated chlorosis (*X. fastidiosa* subsp. *pauca* strain 9a5c), two adjacent, overlapping genes exhibit homology and a syntenic relationship to XatA. The XF1265 protein shows 72% identity to the N-terminal passenger domain of XatA and is predicted to have a signal peptide and four repeat sequences. The XF1264 ORF overlaps the end of XF1265 by 86 bases. The XF1264 protein contains two repeats and the autotransporter domain, but does not appear to have an N-terminal signal sequence. One possible explanation for the absence of XatA in *X. fastidiosa* 9a5c is that a sequencing error introduced a frameshift into the XatA ORF, thereby generating the XF1265 and XF1264 proteins. Indeed, removal of a single nucleotide such as the T residue at position 1,220,709 results in a protein of 807 amino acids. This composite protein exhibits 62% amino acid identity to XatA and is a member of Group I. A more intriguing possibility is that a frameshift mutation has occurred and that *X. fastidiosa* 9a5c is missing XatA. If true, the absence of XatA could have important implications in terms of *X. fastidiosa* 9a5c virulence.

Group II contains orthologs to the *X. fastidiosa* Temecula1 PD1379 protein, which are present in all sequenced *X. fastidiosa* strains. The PD1379 protein, which we have named XatB, has been classified as an AT-1 autotransporter. The XatB and XatA passenger domains are homologous (42% identity) and both contain six copies of 50–60 amino acid repeats. However, the predicted consensus sequence for XatB repeats differs from XatA repeats. This is consistent with our observation that antibodies against the XatA passenger domain do not recognize XatB (data not shown). Group III contains orthologs to the *X. fastidiosa* Temecula1 PD0794 protein. We have named the PD0794 protein XatC based on its membership in the Xat family. Unlike XatA and XatB, XatC is not classified as an autotransporter and its C-terminus does not contain transmembrane-spanning segments or an OMP signature sequence. However, XatC is predicted to have an N-terminal signal sequence. Therefore, if the XatC protein is secreted to the extracellular environment, the mechanism would be different from that used by XatA and XatB.

We also examined the evolutionary relationships between the Xat family and other closely related proteins (Fig. S1). Proteins exhibiting homology to the XatA passenger domain are found in relatively few bacterial species. The closest relatives are found in two species in the β-Proteobacteria, *Neisseria flavescens* and *N. elongata*. Paralogs are also found in two species of the γ-Proteobacteria (*Providencia stuartii* and *Pseudomonas fluorescens* Pf-5) and in a thermohalophilic bacterium in the phylum Bacteriodetes (*Rhodothermus marinus*). We also included in our analysis Cpn0796, an AT-1 autotransporter from *Chlamydia pneumoniae*, which is listed as a putative ortholog of XatA in the OMA database (Altenhoff et al. 2011). However, as shown in Figure S1, there is only weak support for an orthologous relationship between Cpn0796 and XatA.

## Discussion

One of *X. fastidiosa*'s important attributes is its ability to colonize and to form a biofilm within the grapevine xylem and the insect foregut. The initial attachment of bacteria to the host is mediated, in part, by fimbrial and nonfimbrial adhesins (Dunne 2002; MacEachran and O'Toole 2007; Hori and Matsumoto 2010). Although some interactions are nonspecific, the majority are thought to involve specific contacts between adhesins on the bacterial cell surface and receptors located on the surface of the host tissue (Girard and Mourez 2006). The fimbrial adhesins of *X. fastidiosa*, such as the long, filamentous type I pili encoded by *fimA*, have been shown to be involved in biofilm formation and cell–cell aggregation (Feil et al. 2003, 2007; Li et al. 2007; Caserta et al. 2010). Nonfimbrial adhesins have also been implicated in biofilm formation and *X. fastidiosa* pathogenicity. Examples include the trimeric autotransporter XadA (Feil et al. 2007; Caserta et al. 2010), the hemagglutinin adhesins HxfA and HxfB (Guilhabert and Kirkpatrick 2005), and the AT-1 adhesin autotransporter XatA, which is the focus of this manuscript.

The presence of multiple adhesins is a common theme among pathogenic bacteria. This functional redundancy provides the bacterium with the flexibility to recognize many substrates as well as regulate whether or not specific adhesins are present on the cell surface (Dunne 2002; Hori and Matsumoto 2010). Our genetic characterization of *xatA* and our heterologous expression studies allowed us to investigate the contribution of XatA to both biofilm formation and *X. fastidiosa* virulence. These studies revealed that XatA shares many properties with other *X. fastidiosa* nonfimbrial adhesins, such as the hemagglutinins HxfA and HxfB (Guilhabert and Kirkpatrick 2005; Killiny and Almeida 2009b). Like *hxfA* and *hxfB* mutants, the *xatA3* mutant shows reduced biofilm formation and cell–cell aggregation under laboratory conditions. The major difference between XatA and the hemagglutinin mutants is their behavior in grapevines. The *hxfA* and *hxfB* mutants colonize grapevine tissue more rapidly and show earlier symptom development than wild type. Based on this hypervirulent phenotype, HxfA and HxfB have been classified as antivirulence factors (Guilhabert and Kirkpatrick 2005). Their role is to attenuate pathogenicity by limiting the colonization capacity of *X. fastidiosa*, thereby reducing the rate of xylem vessel occlusion. In contrast, the *xatA3* mutant does not colonize grapevine tissue as well as wild type and exhibits few, if any, PD symptoms. Based on these characteristics, XatA has the properties expected for an *X. fastidiosa* virulence factor.

### Proteins homologous to the XatA passenger domain

Homologs to the XatA passenger domain are present in all sequenced *X. fastidiosa* strains and in a few other bacterial species. The *X. fastidiosa* homologs map to a single, well-supported clade, suggesting that they arose from a common ancestor gene. Based on conserved synteny and amino acid sequence, there are three distinct groups of orthologs (XatA, XatB, XatC) within this clade. Orthologs to XatB and XatC are present in all sequenced strains of *X. fastidiosa*. The situation is more complicated in the group containing orthologs to XatA. XatA orthologs are present in all North American isolates of *X. fastidiosa*. However, in the South American isolate *X. fastidiosa* 9a5c, the ORFs of two overlapping genes exhibit homology and a syntenic relationship to XatA. Although the predicted absence of a full-length copy of XatA in *X. fastidiosa* 9a5c could be due to a DNA sequence error, a more intriguing possibility is that *X. fastidiosa* 9a5c does not encode a functional copy of XatA and its absence contributes to the different host range for this particular pathotype.

The absence of a specific autotransporter in some pathotypes is not uncommon. Comparison of 28 sequenced *E. coli* strains revealed that although many autotransporters are present in the majority of strains, others are unique to a particular pathotype (Wells et al. 2010). Some predicted autotransporters, such as YejO, are present in all commensal and diarrheagenic (DEC) genomes, but are truncated in all uropathogenic (UPEC) genomes. Conversely, homologs to the UpaB autotransporter are found in all UPEC strains, but are either missing or truncated in all DEC strains. Similarly, genomic comparison of *C. pneumoniae* isolated from humans and koala uncovered 10 noteworthy regions of single-nucleotide polymorphorisms (SNPs) (Myers et al. 2009). Several of these hot spots encode AT-1 autotransporters, such as the polymorphic outer membrane protein (Pmp) family and the gene cluster containing the Cpn0796 autotransporter (Myers et al. 2009; Mitchell et al. 2010). These strain-specific differences result in antigenic variation, which compromises the host's ability to mount a successful immune response. The presence or absence of specific autotransporters on the cell surface also impacts the ability of the bacterium to interact with the host cell surface, a property shared by both animal and plant pathogens.

### The role of the XatA passenger domain in *X. fastidiosa* virulence

Our fractionation studies indicate that XatA is present in two forms. The first, which includes both the N-terminal passenger domain and the C-terminal β-barrel domain, is the form found in the OM and in OMVs. The second form, which includes just the passenger domain, can be found in the supernatant. The presence of the XatA in the OM is consistent with its function as an adhesin. The XatA passenger domain could facilitate the attachment of *X. fastidiosa* to host tissue, either in the xylem or in the insect foregut or both. This role could explain the properties of the *xatA3* mutant, such as its avirulent phenotype in grapevines. The presence of XatA in OMVs may also have functional significance. OMVs arise naturally from the Gram-negative bacterial OM and have been implicated in biofilm formation and the delivery of virulence factors to host cells (for reviews, see Ellis and Kuehn 2010; Kulp and Kuehn 2010). Therefore, although the presence of XatA in OMVs could simply be a consequence of its presence in the OM during OMV biogenesis, it is also possible that OMVs containing XatA have a role in *X. fastidiosa* pathogenicity.

Another important question concerns the role of cleavage in XatA function. Most AT-1 adhesin autotransporters undergo a cleavage event following their translocation to the cell surface. This event can be autoproteolytic or require an OM-associated protease (for a review, see Girard and Mourez 2006). However, in spite of its prevalence, the importance of this cleavage event for function is not clear. One well-studied example is AIDA-I from diarrheagenic strains of *E. coli*, which plays a role in biofilm formation, autoaggregation, and pathogenicity. After translocation, AIDA-I undergoes autocatalytic cleavage and the resulting mature product remains tightly and noncovalently associated with the C-terminal domain (Charbonneau et al. 2009). Interestingly, studies using an uncleavable point mutant of AIDA-I revealed that the proteolytic processing of AIDA-I does not appear to be important for its function under laboratory conditions (Charbonneau et al. 2006). In contrast, autoproteolytic cleavage of the *Haemophilus influenzae* Hap autotransporter is thought to have an important regulatory role in pathogenicity (Fink and St Geme 2003; Kenjale et al. 2009). The Hap passenger domain has serine protease activity and is responsible for mediating adhesion to epithelial cells. However, only the uncleaved, cell-associated form of the protein has a role in adherence (Hendrixson and St Geme 1998). Moreover, inhibitors and mutations that prevent the release of the Hap passenger domain result in increased adherence to epithelial cells and autoaggregation. Therefore, controlling the autoproteolytic release of the Hap passenger domain from the cell surface allows the bacterium to modulate its interactions with the host tissue during different stages of the infection. Based on our fractionation studies, the cleaved XatA passenger domain is not tightly associated with the OM and can be found free in the supernatant. More experiments are needed to determine if the released form of XatA is functional and how inhibitors or mutations that prevent XatA cleavage impact *X. fastidiosa* virulence. The insights gained from these experiments could result in the development of new therapies to mitigate the development of PD of grapevines.

## Experimental Procedures

### Bacterial strains, plasmids, and growth conditions

The key *X. fastidiosa* and *E. coli* strains and plasmids used in this study are listed in Table 1. A complete list of the bacterial strains and plasmids is presented in Table S1. *Xylella fastidiosa* strains were grown at 28°C on PD3 medium (Davis et al. 1981). The antibiotic concentrations used to generate mutants and to maintain plasmids in *X. fastidiosa* were as follows: chloramphenicol 5 μg mL^−1^ and gentamicin 5 μg mL^−1^. Growth rates, autoaggregation, and quantification of biofilm formation of *X. fastidiosa* strains were determined as previously described (Matsumoto et al. 2009). *E. coli* strains were grown at 37°C on Luria–Bertani (LB) medium (Sambrook and Russell 2001). The antibiotic concentrations used to maintain plasmids in *E. coli* were as follows: ampicillin 100 μg mL^−1^, chloramphenicol 25 μg mL^−1^, gentamicin 10 μg mL^−1^, and kanamycin 50 μg mL^−1^.

### DNA manipulation techniques and primers

Standard recombinant DNA procedures were employed (Sambrook and Russell 2001). The primers used in this study are listed in Table S2. Depending on the vector used, intermediate plasmids were first introduced by electroporation into DH5α, TOP10, or EAM1 (Table S1). Plasmid DNA was isolated from *E. coli* using a miniprep kit (Qiagen Inc., Valencia, CA, USA); the amount of DNA was quantified by measuring absorbance at 260 nm using a NanoDrop 1000 spectrophotometer (NanoDrop Technologies, Wilmington, DE, USA). PCR-generated inserts in constructed plasmids were subjected to DNA sequence analysis at UC Davis DNA Sequencing Facility. Preparation of *X. fastidiosa* competent cells and the conditions for electroporation were according to previously published procedures (Matsumoto et al. 2009; Matsumoto and Igo 2010).

### Isolation of OM proteins, OMVs, and OM-secreted proteins from *X. fastidiosa*

To prepare the liquid PD3 cultures, *X. fastidiosa* cells grown on PD3 plates were suspended with phosphate-buffered saline (PBS; pH7.4) (Sambrook and Russell 2001). The cell suspension was added to 25 or 30 mL of liquid PD3 medium and grown for seven days. The culture was then added to 1.0 L of PD3 medium and incubated with shaking (100 rpm) at 28°C. The cells and supernatant fraction were separated by centrifugation at 5000 *g* for 15 min at 4°C. The OM fraction was separated from other cellular components using a sucrose step gradient as previously described (Voegel et al. 2010). The OM fraction was diluted with 10 mM HEPES pH 7.4 and recovered by centrifugation at 100,000 × *g* for 1 h at 4°C. The pellet was resuspended in 100 μl of 10 mM HEPES pH 7.4 with 0.1 mM PMSF and 0.1 M EDTA. The proteins present in the pellet were designated as OMPs. To obtain the membrane vesicles and the secreted proteins, the supernatant fraction was vacuum filtered through a 0.45 μM followed by a 0.2 μM pore size filter (Millipore, Billerica, MA, USA) and subjected to centrifugation at 38,000 × *g* for 1 h at 4°C. An aliquot of the 38,000 × *g* supernatant (100 mL) was then centrifuged at 150,000 × *g* for 3 h at 4°C. The supernatants from this spin were concentrated approximately 100-fold using a Centricon Plus-70 (Millipore; molecular cutoffs, 10,000) according to the manufacturer's instruction. The concentrated supernatant fraction was designated as the secreted proteins; the pellet was designated as the OMV fraction. Protein concentrations in the fractions were determined by using a BCA protein assay kit (Pierce Chemicals, Rockford, IL, USA) according to the manufacturer's instruction.

### Assignment of XatA to a specific gene on the *X. fastidiosa* chromosome

To assign OMPs to specific genes on the *X. fastidiosa* chromosome, proteins in the OM fraction were separated by SDS-PAGE and stained with either SyproRuby (Biorad, Hercules, CA, USA) or Coomassie Blue stain. Well-isolated bands were excised and the proteins in each band were subjected to trypsin digestion. The protein fragments were then analyzed by matrix-assisted laser desorption/ionization–time-of-flight (MALDI-TOF) mass spectrometry at the UC Davis Molecular Structure Facility and the resulting information was compared to proteins in the Swiss-Prot and NCBInr databases using either MASCOT at http://www.matrixscience.com or MS-Fit at Protein Prospector (UCSF; http://prospector.ucsf.edu). One of the proteins mapped to the PD0528 locus and was named XatA.

### Preparation of rabbit antiserum against purified XatA passenger domain

The DNA encoding the XatA passenger domain (amino acid 29 to 470) was amplified with primers PD0528–29 and PD0528–470 using *X. fastidiosa* Temecula1 as the template. The 1.34-kb PCR product was cloned into pCR-Blunt II-TOPO resulting in plasmid pAM53. The *xatA* insert in pAM53 was removed by digestion with NdeI and XhoI, purified by gel extraction, and ligated into the NdeI and XhoI sites of pET-29b (Novagen, Merck KGaA, Darmstadt, Germany). The resulting plasmid pAM54 was introduced into *E. coli* strain BL21(DE3) by electroporation. To obtain purified XatA passenger domain, BL21(DE3) harboring pAM54 was grown in 100 mL LB containing 50 μg mL^−1^ kanamycin to an optical density at OD_600_ of 0.6. Expression of the XatA passenger domain was induced for 2 h with 1 mM IPTG and the cells were harvested by centrifugation. The His-tagged recombinant protein (RXatA) was isolated by affinity purification using an Ni-NTA slurry (Qiagen) under denaturing conditions according to the manufacturer's instruction. Purified RXatA was then sent to the Comparative Pathology Laboratory at UC Davis where it was injected into New Zealand white rabbits to raise a polyclonal antibody against the XatA passenger domain (αRXatA).

### Immunoblot analysis

After suspension in 1% SDS/65 mM Tris HCl, pH 7.0, proteins in the different fractions were resolved by SDS-PAGE and transferred to a Sequi-Blot polyvinylidene difluoride (PVDF) membrane with a Mini Trans-Blot cell using the protocol provided by the manufacturer (Bio-Rad). The blots were probed with a 1:500 dilution of αRXatA antibody, washed five times, and then probed with goat anti-rabbit secondary antibody conjugated to horseradish peroxidase (Pierce Chemicals). Bound conjugate was detected using the ECL detection reagents (GE Healthcare Biosciences, Piscataway, NJ, USA) according to manufacturer's instructions and the images were captured using a FluorChem Imaging system (Alpha Innotech, San Leanardo, CA, USA).

### Protease accessibility assays

To determine the protease accessibility of XatA, a 200-mL PD3 culture of *X. fastidiosa* was prepared and divided in two 100-mL aliquots. The cells were harvested by centrifugation at 3,000 × *g* for 15 min at 4°C and suspended in 10 mM Tris-HCl, pH7.6, containing 10 mM MgCl_2_. The cell suspension from one aliquot was incubated with Proteinase K (Sigma-Aldrich, St. Louis, MO, USA) at a concentration of 10 μg mL^−1^ at 37°C for 30 min. The second aliquot was not treated and served as a control. PMSF (final concentration 5 mM) was then added to the samples to inhibit further proteolysis. The cells were harvested and the OMPs were isolated using a linear sucrose step gradient as described above. The proteins were then separated by SDS-PAGE and the presence of the XatA passenger domain was determined by immunoblotting using αRXatA.

### Construction of the *xatA3* mutant and the complementation strain

To generate the *xatA3* mutant, a 2.65-kb fragment containing the *xatA* ORF and flanking sequences was amplified from Temecula1 genomic DNA using primers PD0528F-Spe and PD0528R-Spe. The resulting PCR products were cloned into pCR-Blunt II-TOPO (Invitrogen, Carlsbad, CA, USA), generating pAM34. The 1.14-kb fragment containing a chloramphenicol-resistance gene was then amplified from pRL1342 (Wolk et al. 2007) with primers Cm-f and Cm-r, digested with SpeI, and ligated into the unique NheI site located within the *xatA* insert on pAM34 to generate pAM109. The insertion into NheI disrupts the *xatA* opening reading frame at amino acid 294. Plasmid pAM109 was introduced into *X. fastidiosa* by electroporation and transformants were selected on PD3 plate supplemented with 5 μg mL^−1^ chloramphenicol. Transformants were streaked onto fresh PD3 selective plates and grown for seven to 10 days. The colonies were then screened by PCR to identify transformants that carried the chloramphenicol cassette at *xatA* and were missing the wild-type *xatA* allele. One of the transformants containing the *xatA3*::Cm^r^ mutation was selected for further study and named TAM103.

For genetic complementation analysis of the *xatA3*::Cm^r^ mutation, a 3.5-kb fragment containing *xatA* was amplified using primers PD0528_fwd and PD0528_rev and inserted into pCR-BluntII-TOPO, generating pLBT0528. The fragment carrying the *xatA* gene was removed using SpeI and XbaI, and then inserted into the unique XbaI site in pBBR1MCS-5. The resulting plasmid, pAM61, was introduced into TAM103 by electroporation, and transformants were selected on PD3 supplemented with 5 μg mL^−1^ gentamicin. The presence of pAM61 in the transformants was confirmed by PCR using primers specific for the pBBR1 replicon and the *xatA* gene. One of the transformants was designated as TAM103/pAM61 (*xatA3*/p-*xatA*^+^).

### Heterologous expression of XatA in *E. coli* strain UT5600

For this analysis, pAM61 (p-*xatA*^+^) and pBBR1MCS-5 (vector) were introduced into UT5600 (Elish et al. 1988; Kaufmann et al. 1994). To confirm the localization of XatA to the *E. coli* cell surface, the OMPs were isolated using the method described by Morona and Reeves (1982). Overnight cultures of UT5600/pBBR1MCS-5 and UT5600/pAM61 were also subjected to the protease accessibility assay described above. The proteins in the OM fraction were then analyzed by SDS-PAGE and immunoblotting with αRXatA.

To examine autoaggregation, UT5600/pAM61 and UT5600/pBBR1MCS-5 cultures were concentrated to an OD_600_ of 2.5 in 5 mL LB in 16 × 125 mm borosilicate tubes. The cultures were vortexed for 10 sec and incubated statically for 3.5 h at room temperature. Samples of 100 μl were taken from the top of the tubes at 30-min intervals and the OD_550_ was determined. Each experiment was performed in triplicate. The ability of the strains to generate a biofilm was detected by staining with crystal violet (O'Toole and Kolter 1998). Briefly, 100 μl of the cultures were added to 1 mL LB broth containing gentamicin and grown at 30°C for two days in 18-mm glass tubes without agitation. Then, 100 μl of 0.1% crystal violet was added and the tubes were incubated for 30 min at room temperature. The medium was removed and the attached cells were rinsed three times with distilled water. The stained biofilms were eluted with 1 mL of 95% ethanol and the OD_570_ was measured to quantify the extent of biofilm formation. Each experiment was performed in triplicate.

### Pathogenicity assay on grapevines

*Xylella fastidiosa* Temecula1 and TAM103 (*xatA3*::Cm^r^) were inoculated individually into two-month-old greenhouse-grown grapevines (cv. Thompson seedless) by the needle puncture method (Hill and Purcell 1995). Each plant was inoculated twice with ∼10^7^ cells at a position two internodes up from the base of the stem. As a negative control, a mock inoculum of water was also prepared. This experiment was performed in triplicate for each inoculum. In an independent series of experiments, grapevines were inoculated twice with ∼10^6^ cells (TAM22, TAM103, or TAM103/pAM61) at a position two internodes up from the base of the stem. As a negative control, a mock inoculum of PBS (pH 7.4) was also prepared. This experiment was performed in triplicate for each inoculum.

After 24 weeks, petiole tissue was sampled at various distances above the inoculation point and *X. fastidiosa* population at each location was determined by plating on PD3 plates as previously described (Matsumoto et al. 2009). *Xylella fastidiosa* isolated from the TAM103/pAM61-inoculated vines were also plated on PD3 plates with 5 μg mL^−1^ gentamicin to determine whether or not the plasmid was lost during the infection.

### Insect transmission

The blue-green sharpshooters *G. atropunctata* (Signoret) (Hemipetra: Cicadellidae) used in this study were reared on sweet basil. To ensure that the sharpshooters were *X. fastidiosa*-free (*X. fastidiosa* is not transmitted transovirally), we used second-generation adults. Insect transmissibility was examined using the method described by Killiny and Almeida (2009a; Killiny et al. 2012). Briefly, *X. fastidiosa* cells were grown for seven days on XFM-pectin medium and then suspended in an artificial diet solution to a final concentration of 10^8^ cfu mL^−1^. Individual blue-green sharpshooters were allowed access to the sachet containing the bacteria and diet solution for 3 h. Insects were individually caged on a single leaf of a grape host, using a small 2-cm (in diameter) clip cage and were allowed to feed for a four-day inoculation access period at 20°C. After four days, the insects were removed and each corresponding leaf was marked. Twenty-five replicates were performed for each strain. After 12 weeks, the petioles of the marked leaves were examined for the presence of *X. fastidiosa* using standard culturing protocols (Hill and Purcell 1995). The percentage of infected to healthy leaves was compared for the four strains of *X. fastidiosa* using one to four contingency table analysis followed by pair-wise comparisons using Fisher's exact test (∞ = 0.05) with Bonferroni's correction to count multiple comparisons.

### Computer analysis of XatA

The homology of XatA to autotransporter proteins was initially discovered using BlastP (Altschul et al. 1990) and the domains were detected using the Prodom (Bru et al. 2005) and Pfam (Finn et al. 2008) databases. The signal peptide of XatA was predicted using the SignalP3.0 program (Emanuelsson et al. 2007) and the presence of the C-terminal OMP signature sequence was predicted by comparing the last 15 amino acids of XatA to the last 15 amino acids of both the *E. coli* and *X. fastidiosa* OMPs listed in the OMPdb database (Tsirigos et al. 2011). Secondary structure predictions for the XatA passenger domain were obtained using PsiPred (McGuffin et al. 2000). Further analysis of this domain was conducted using HHrepID, a method for the de novo identification of highly diverged protein repeats (Biegert and Soding 2008). This led to the identification of six potential repeats. A consensus sequence for the six central repeats was then generated using multiple alignment programs, such as ClustalW2.1 (Chenna et al. 2003) and Multalin (Corpet 1988).

### Identification of homologs to the XatA passenger domain

PSI-BLAST (Altschul et al. 1997) was used to search the NCBI database containing nonredundant protein sequences (nr) for proteins exhibiting homology to amino acids 30–426 of XatA. Sequences with *E*-values above 2.00E-05 were aligned with MUSCLE (http://www.biomedcentral.com/1471-2105/5/113) and visually screened in Jalview to remove redundancies and fragmentary sequences. The resulting pool of sequences was used to generate 1000 maximum likelihood bootstrapped phylogenetic trees using RAxML with the JTT substitution model (http://bioinformatics.oxfordjournals.org/content/22/21/2688.long) and visualized using FigTree.

Although the Texas isolate *X. fastidiosa* subsp. *fastidiosa* GB514 does not contain a homolog to XatA in the current annotation, its absence is most likely due to sequencing errors in two adenine-rich regions, which result in a frameshift. In *X. fastidiosa* subsp. *pauca* 9a5c, *xatA* (PD0528) shows a syntenic relationship with two loci, XF1264 and XF1265. The composite protein XF1265/XF1264 was generated by the removal of a base pair at position 1,220,709, which is close to the stop codon at the end of XF1265. This composite protein was included in the phylogenetic analysis presented in Figure S1.

## Supporting information

**Figure S1.** Phylogenetic tree of XatA homologs.

**Table S1.** Bacterial strains and plasmids used in this study.

**Table S2.** Primers.
