# Supplementary material for: XatA, an AT-1 autotransporter important for the virulence of Xylella fastidiosa Temecula1
Source: Microbiologyopen. 2012 Mar;1(1):33–45. doi: 10.1002/mbo3.6 (PMC3426408; doi:10.1002/mbo3.6)
Supplement: Supplementary file 1 [file mbo30001-0033-SD1.pdf]

Table S1. Bacterial strains and plasmids used in this study

Strains or plasmids	Description	Source
Xylella fastidiosa subsp. *fastidiosa*		
Temecula1	Wild type, Pierce's disease strain	(Guilhabert *et al.*, 2001)
TAM22	Temecula1, NS1::Cm^{r}	(Matsumoto *et al.*, 2009)
TAM103	Temecula1, *xatA3*::Cm^{r}	This study
TAM103/pAM61	TAM103 with pAM61- Cm^{r}, Gm^{r}	This study
Escherichia coli		
BL21(DE3)	F^{-} *dcm ompT hsdS*$_{B}$(r$_{B}^{-}$ m$_{B}^{-}$) *gal* (λDE3)	Novagen
EAM1	DH5α derivative; Sp^{r} St^{r} *attP*$_{HK022}$::(P$_{LlacO-1}$ -PD1607)	(Matsumoto and Igo, 2010)
DH5α	F^{-} *endA1 recA1 gyrA96 thi-1 hsdr17(r*$_{K}^{-}$ *m*$_{K}^{+}$*) relA1 supE44* Δ*(lacZYA-argF)U169 Φ80lacZ*Δ*M15 deor phoA λ*$^{-}$	Lab collection
TOP10	F^{-} *mcrA* Δ(*mrr-hsd*rMS-*mcrBC*) Φ80*lacZ*ΔM15 Δ*lacX*74 *recA*1 *araD*139 Δ(*ara-leu*)7697 *galU galK rpsL* (St^{r}) *endA*1 *nupG*	Invitrogen
UT5600	F- *ara-14 leuB6 azi-6 lacY1 leu-6 proC14 tsx-67 entA403 trpE38 rfbD1 rpsL109 xyl-5 mtl-1 thi1* Δ(*ompT-fepC266)* Δ*ompP*	(Elish *et al.*, 1988; Kaufmann *et al.*, 1994)
Plasmids		
pAM34	*xatA*$^{+}$ (2.65 kb) in pCR-Blunt II-TOPO, Km^{r}	This study
pAM53	*xatA* passenger domain in pCR-Blunt II-TOPO, Km^{r}	This study
pAM54	*xatA* from pAM53 in pET-29b, Km^{r}	This study
pAM61	*xatA*$^{+}$ (3.5 kb) in pBBR1MCS-5, Gm^{r}	This study
pAM109	*xatA3*::Cm^{r} in pCR-Blunt II-TOPO; Cm^{r}, Km^{r}	This study
pBBR1MCS-5	Broad-host range cloning vector, Gm^{r}	(Kovach *et al.*, 1995)
pCR-Blunt II-TOPO	Blunt PCR cloning vector, Km^{r}	Invitrogen
pET-29b(+)	Overexpression vector, Km^{r}	Novagen
pLBT0528	*xatA* (3.5 kb) in pCR-Blunt II-TOPO	This study
pRL1342	RSF1010 replicon; Cm^{r}, Em^{r}	(Wolk *et al.*, 2007)

Table S2: Primers

**Primer name**	**Primer sequence (5'→3')**	**Restriction Sites**	**Source or reference**
For generating of *xatA* mutant and complementation			
Cm-f	GGACTAGTCCAATCAGCGACACTGAATACGG	SpeI	(Matsumoto *et al*., 2009)
Cm-r	GGACTAGTCCTCACTTATTCAGGCGTAGCAC	SpeI	(Matsumoto *et al*., 2009)
PD0528F-Spe	CAAACTAGTACAAGAACATGACGTGACGC	SpeI	This study
PD0528R-Spe	CAGACTAGTAACAGCGCTATTGGGCGATT	SpeI	This study
PD0528_fwd	CGCCAACGTTTATTCGAATTGCC		This study
PD0528_rev	GAGTAGTTGCGCAGCTTAGACTTTCATCC		This study
For generating the XatA passenger domain for antibody production			
PD0528-29	CATATGAACTCGCCATCCATATCGGAG	NdeI	This study
PD0528-470	CTCGAGCGCGTCTATCTGACAGTGTTG	XhoI	This study

2

Figure S1.

Phylogenetic tree of XatA homologs.

Sequences exhibiting homology to the XatA passenger domain (amino acids 30-426) with E-values above 2.00E-05 were aligned with MUSCLE (http://www.biomedcentral.com/1471-2105/5/113) and visually screened in Jalview to remove redundancies and fragmentary sequences. The resulting pool of sequences was used to generate 1000 maximum likelihood bootstrapped phylogenetic trees using RAxML with the JTT substitution model (http://bioinformatics.oxfordjournals.org/content/22/21/2688.long) and visualized using FigTree. Numbers by nodes are bootstrap values. The *X. fastidiosa* Temecula1 homologs are in gray. The followings proteins were used for the analysis: XatA orthologs [Xf Temecula-XatA (NP_778752.1), Xf M23-XatA (YP_001829270.1), Xf M12-XatA (YP_001775231.1), Xf Dixon-XatA (ZP_00651219.1), Xf Ann1-XatA (ZP_00682978.1), Xf CVC-XF1265/XF1264 (composite protein generated from DNA sequence by removing a single nucleotide at position 1,220,709, which results in a protein of 807 amino acids.)]; XatB orthologs [Xf Temecula1-XatB (NP_779577.1), Xf Ann1-XatB (ZP_00680532) , Xf GB514-XatB (ADN62223.1), Xf CVC-XatB (NP_299628.2), Xf Dixon-XatB (ZP_00652420.1)]; XatC orthologs [Xf Temecula1-XatC (NP_779013.2), Xf M23-XatC (YP_001829549.1), Xf GB514-XatC (ADN63803.1), Xf Ann1- XatC (ZP_00680178.1), Xf CVC-XatC (NP_299303.2), Xf Dixon-XatC (ZP_00652050.1), Xf CVC-XF2069 (NP_299348.1)]: Other bacteria **[**NEIELOOT_00780 (*Neisseria elongata* subsp. *glycolytica*; ZP_06733955.1), NEIFL0001_2154 (*Neisseria flavescens* SK114; ZP_04757153.1), PROSTU_00583 (*Providencia stuartii* ATCC 25827; ZP_02958823.1), PFL_2727 (*Pseudomonas fluorescens* Pf-5; YP_259834.1), Rmar_2382

3

(*Rhodothermus marinus* DSM 4252; YP_003291648.1), AGROH133_04441 (*Agrobacterium* sp. H13-3; YP_004278026), CPn0796 (*Chlamydia pneumoniae* CWL029; NP_224991.1)].

4

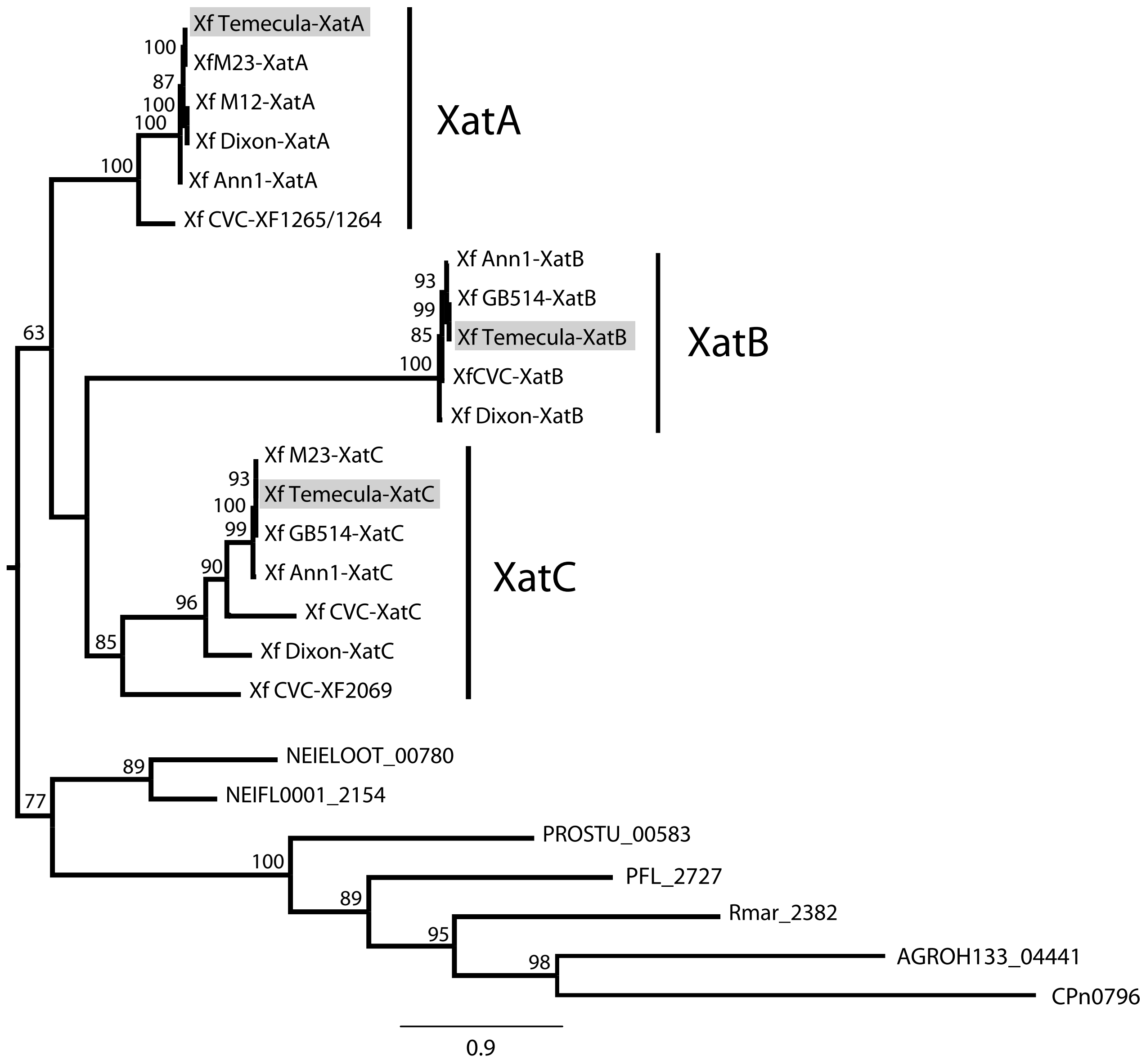

Xf Temecula-XatA
XfM23-XatA
Xf M12-XatA
Xf Dixon-XatA
Xf Ann1-XatA
Xf CVC-XF1265/1264
XatA
Xf Ann1-XatB
Xf GB514-XatB
Xf Temecula-XatB
XfCVC-XatB
Xf Dixon-XatB
XatB
Xf M23-XatC
Xf Temecula-XatC
Xf GB514-XatC
Xf Ann1-XatC
Xf CVC-XatC
Xf Dixon-XatC
Xf CVC-XF2069
XatC
NEIELOOT_00780
NEIFL0001_2154
PROSTU_00583
PFL_2727
Rmar_2382
AGROH133_04441
CPn0796
100
87
100
100
100
63
93
99
85
100
93
100
99
90
96
85
89
77
100
89
95
98
0.9
