# Supplementary figures and images for: XatA, an AT-1 autotransporter important for the virulence of Xylella fastidiosa Temecula1
Source: Microbiologyopen. 2012 Mar;1(1):33–45. doi: 10.1002/mbo3.6 (PMC3426408; doi:10.1002/mbo3.6)

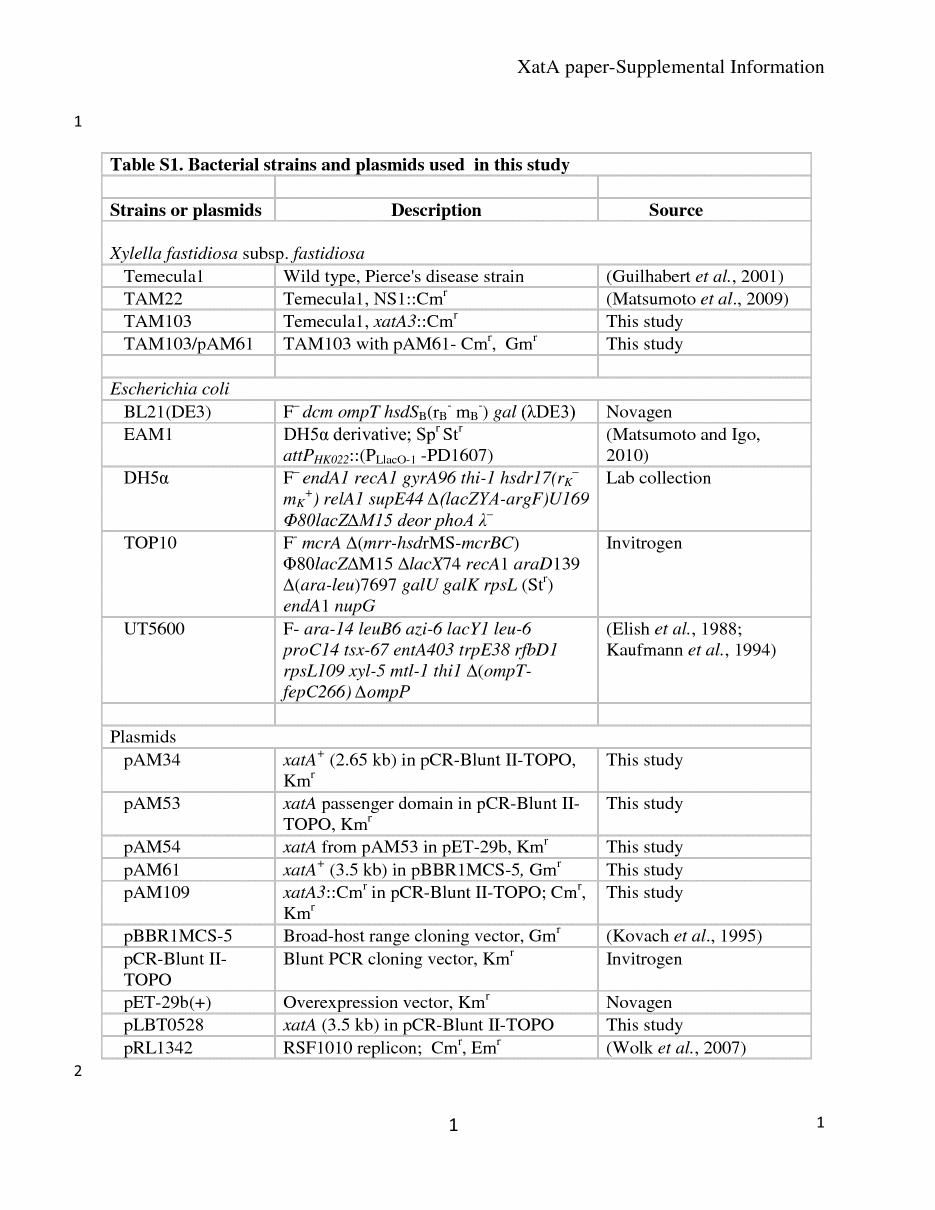

Supplement: Supplementary file 2 [file mbo30001-0033-SD2.png]
